# Supplementary material for: Optimal Examination Sites for Periodontal Disease Evaluation: Applying the Item Response Theory Graded Response Model
Source: J Clin Med. 2020 Nov 21;9(11):3754. doi: 10.3390/jcm9113754 (PMC7700480; doi:10.3390/jcm9113754)
Supplement: Supplementary file 1 [file jcm-09-03754-s001.zip › materials/Figure_S1-S4.docx]

(A)


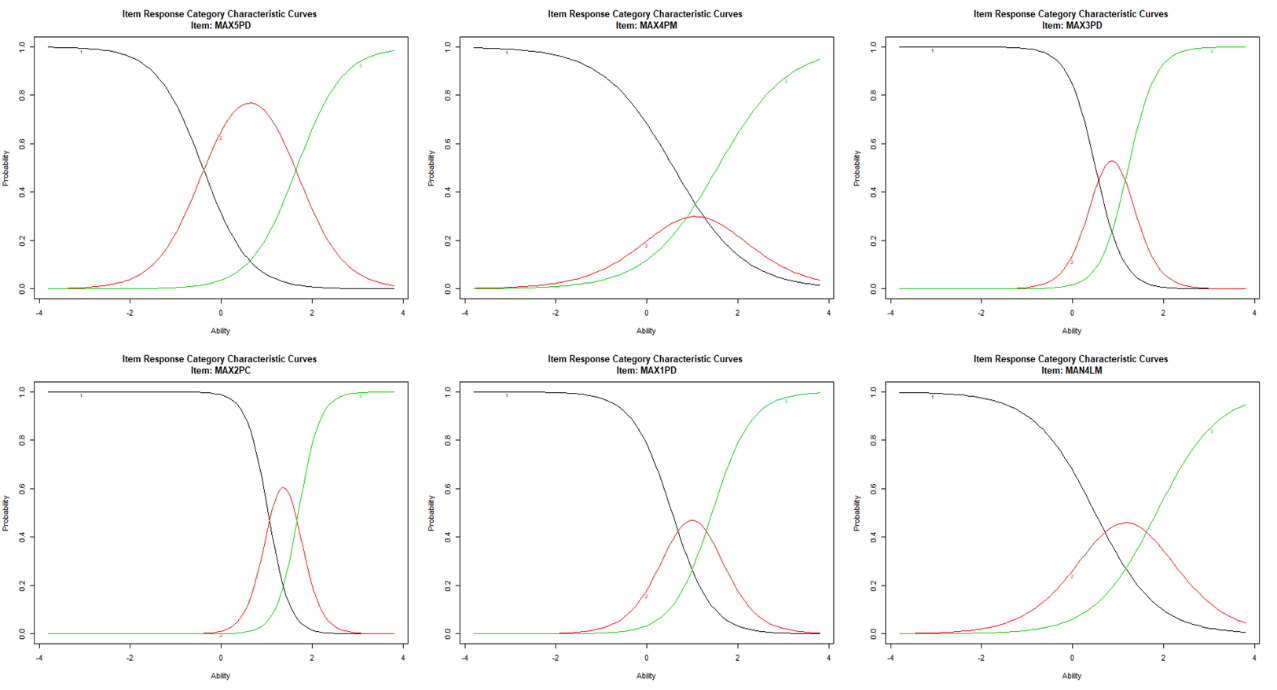


(B)


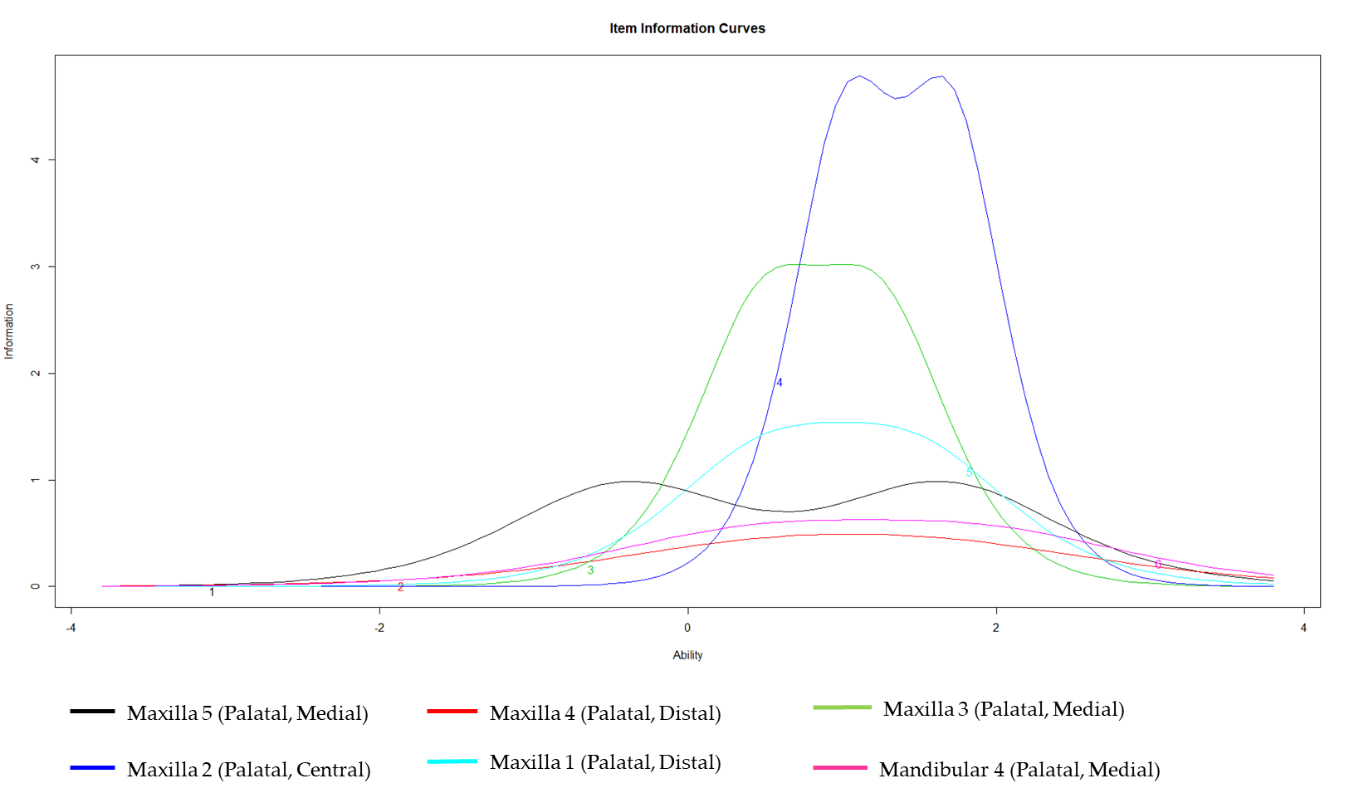


**Figure S1.** Item information curves and item response curves for the six selected values of the clinical attachment level

1. Item response curve

Black line: item information curve of CAL <4mm

Red line: item information curve of CAL 4−5 mm

Green line: item information curve of CAL >=6mm

CAL: clinical attachment level

1. Item information curve

Among the selected sites with high item information, the maxillary canine and lateral incisor had the highest information.


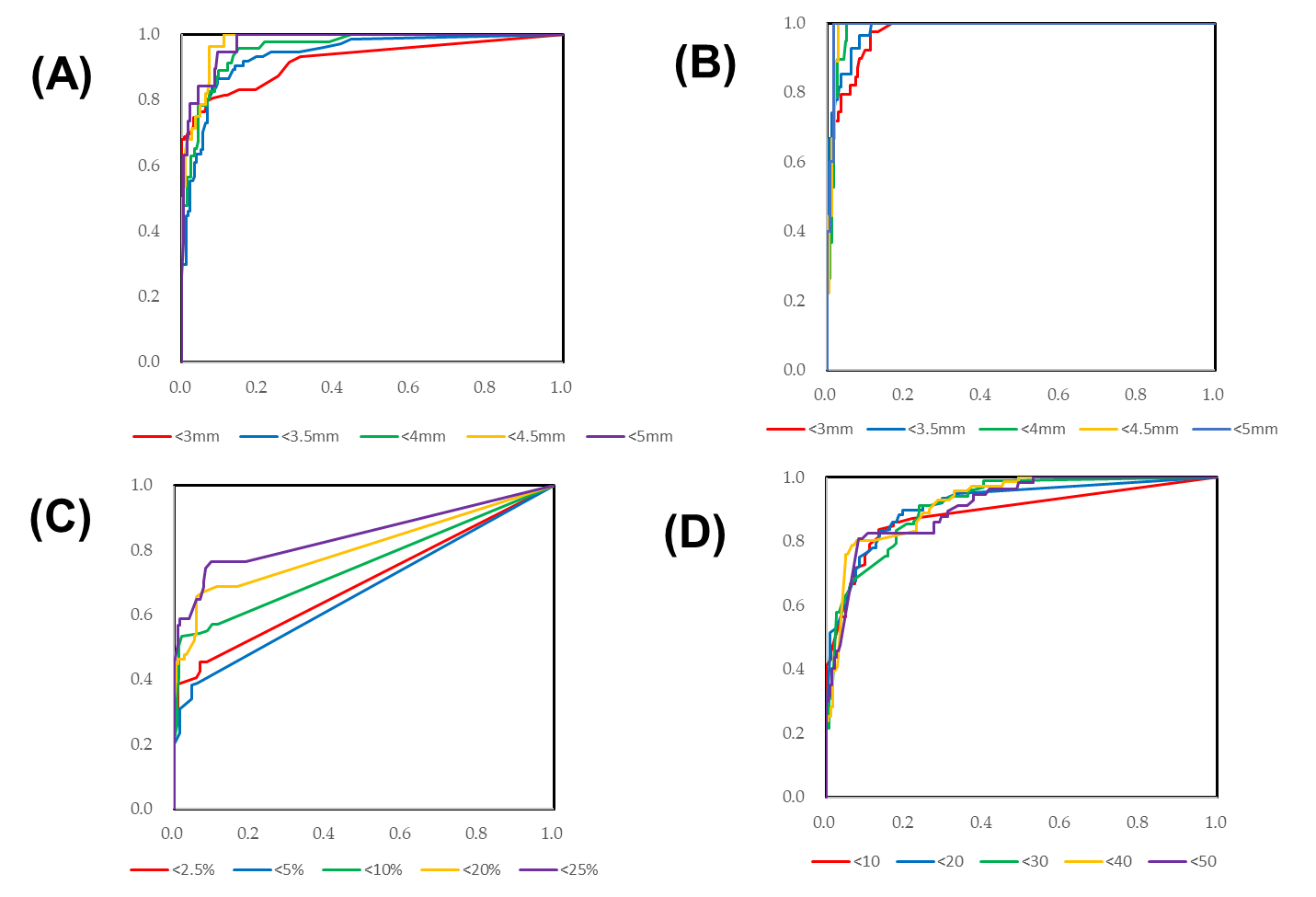


**Figure S2.** Receiver operating characteristic curves for the six selected values

1. Clinical attachment level, (B) Probing pocket depth, (C) Bleeding on probing, and (D) Plaque Index.


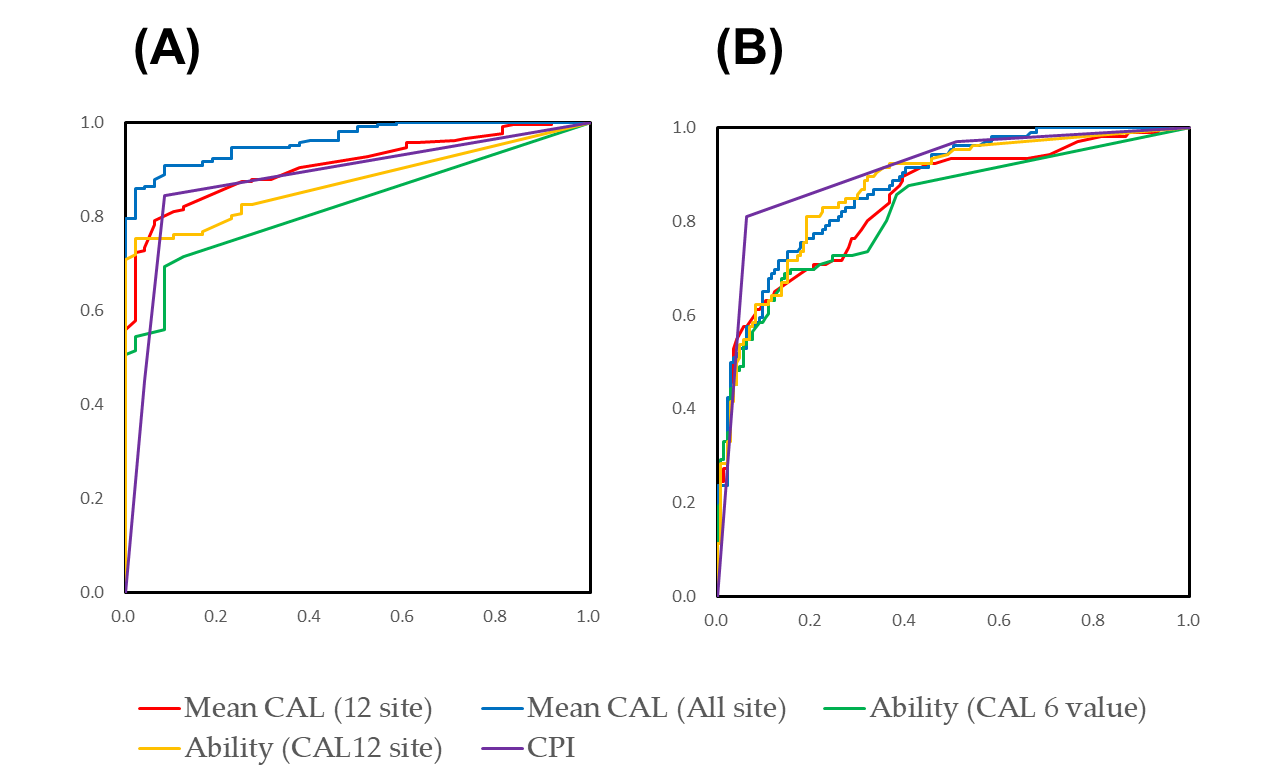


**Figure S3.** Receiver operating characteristic curves for the diagnosis of periodontal disease by the selected sites

1. Moderate periodontitis (B) Severe periodontitis

Diagnostic criteria proposed by the Center for Disease Control and Prevention in partnership with the American Academy of Periodontology was used as gold standard.


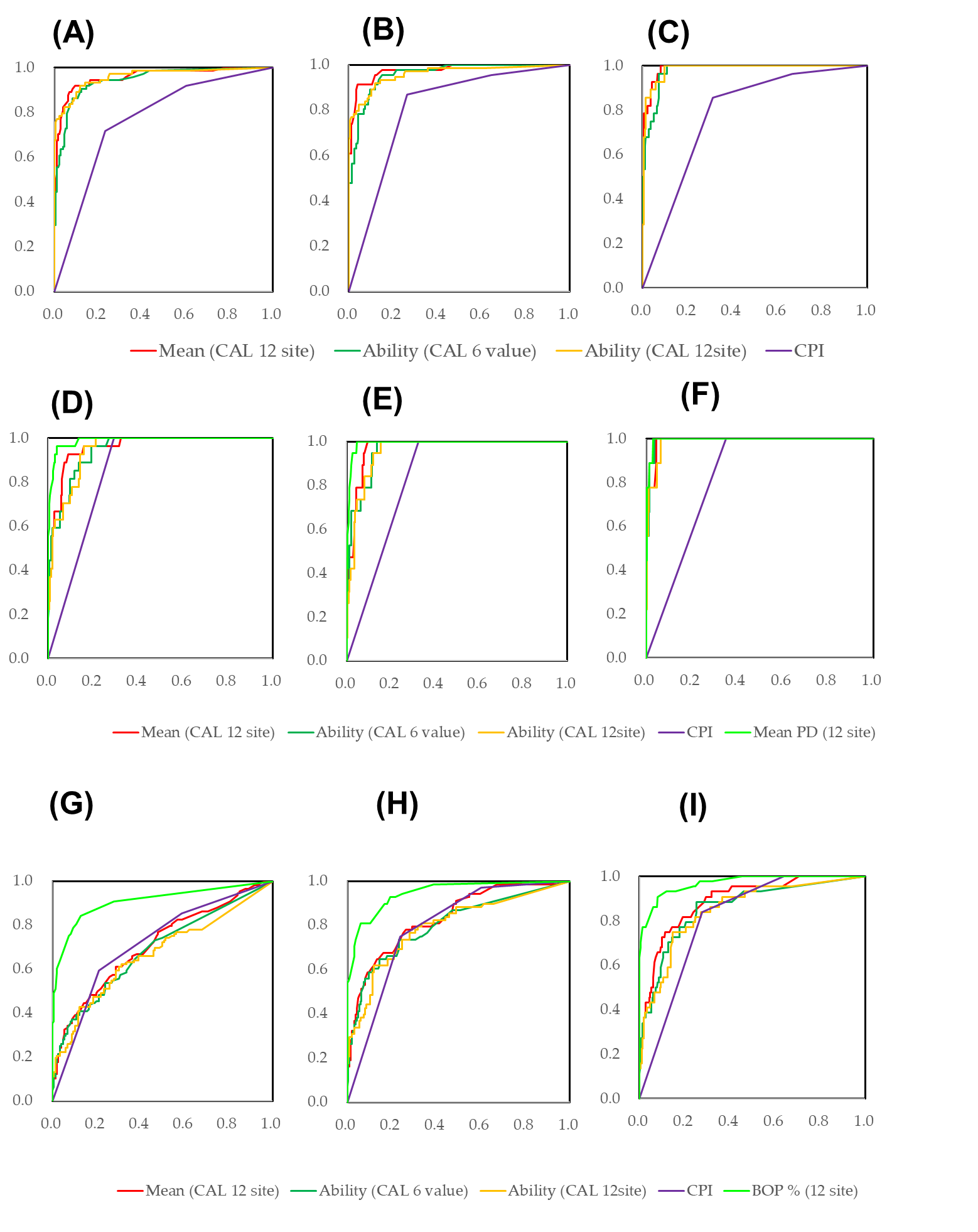


**Figure S4.** Receiver operating characteristics curve for the prediction of clinical parameters by the selected site

1. Mean CAL > 3.5 mm, (B) Mean CAL > 4 mm, (C) Mean CAL > 4.5 mm (D) Mean PD > 3.5 mm, (E) Mean PD > 4 mm, (F) Mean PD > 4.5 mm, (G) BOP% >10%, (H) BOP% >20%, (I) BOP% >30%

Cutoff values are shown in Table 4.
